# Association between executive functions and fear of falling among people aged 80 years or older: a cross-sectional study

**DOI:** 10.1186/s12877-025-06067-5

**Published:** 2025-06-02

**Authors:** Anna Awad, Anna Sundström, Felicia Gramner, Ursula Werneke, Annika Toots, Birgitta Olofsson, Albin Dahlin Almevall, Erik Rosendahl, Stefan Söderberg, Yngve Gustafson, Johan Niklasson

**Affiliations:** 1 Department of Community Medicine and Rehabilitation, Geriatric Medicine, Sunderby Research Unit, Umeå University, 901 87 Umeå, Sweden; 2 Department of Public Health and Clinical Medicine, Umeå University, Umeå, Sweden and Department of Health, Education, and Technology, Luleå University of Technology, Luleå, Sweden; 3 Department of Clinical Sciences, Sunderby Research Unit, Umeå University, Psychiatry Umeå, Sweden; 4 Department of Community Medicine and Rehabilitation, Physiotherapy, Umeå University, Umeå, Sweden; 5 Department of Nursing, Department of Diagnostics and Interventions, Umeå University, Umeå University, Umeå Orthopedics, Sweden; 6 Department of Public Health and Clinical Medicine, Umeå University, Umeå, Sweden; 7 Department of Public Health and Clinical Medicine, Medicine, Umeå University, Umeå, Sweden; 8 Department of Community Medicine and Rehabilitation, Geriatric Medicine, Umeå University, Umeå, Sweden

**Keywords:** Aged, 80 and over, Executive function, Fear of falling, FAB, FES-I

## Abstract

**Objectives:**

Fear of falling (FoF) is a common problem among older adults. It can lead to reduced quality of life and less physical activity, which increases fall risk. Earlier work has shown that FoF can be a manifestation of executive dysfunction in adults over 50 years, but studies on people over age 75 years are lacking. Executive functions (EFs) are cognitive functions associated with the frontal lobes and the prefrontal cortex. The aim of this study was to assess associations of EFs and FoF among people aged 80 years or older.

**Methods:**

This cross-sectional study was based on data from the Northern Sweden Silver-MONICA study and included 434 participants aged 80 years or older. EFs were assessed with the Frontal Assessment Battery (FAB) and FoF with the Falls Self-Efficacy Scale–International (FES-I). Multivariable linear regression analysis was used to examine associations among EF, FoF, and a comprehensive set of adjustment factors. Pearson correlation analysis was used to evaluate associations of FES-I and the subitems of the FAB.

**Results:**

EFs as measured by FAB were inversely associated with FoF (β = -0.23; 95% confidence interval, -0.42 to -0.03; *p* = 0.021), even after comprehensive adjustments. The FAB subitems measuring lexical fluency, inhibitory control, sustained attention, self-organization, motor programming, and planning also were inversely associated with FoF.

**Conclusions:**

Lower EF is associated with higher FoF among people aged 80 years or older. This information is important for treating and preventing FoF in this population.

## Background

Fear of falling (FoF) is common among older adults [1] whether they have experienced a fall or not. Prevalence estimates range from 20 to 39% overall [2] and from 40 to 73% among those who have experienced falls [3]. It is crucial to understand, treat, and prevent FoF, which can lead to reduced quality of life and less physical activity [4], contributing to adverse health outcomes such as physical decline [5], frailty [6], and increased fall risk [7]. Approximately one-third of people over age 65 years fall every year, and the number is higher among those living in group settings, such as elder care [8]. Falls can be reduced by physical exercise that improves muscle strength and balance [9–11].

FoF has been defined as an emotional response to a real or imagined threat to balance, but the term typically encompasses more generalized concerns about falling [12], According to MacKay et al. [13], factors consistently associated with FoF are older age, female sex, previous falls, poor physical performance, and symptoms of depression. Age-related slower gait speed and poorer balance, both of which contribute to falls and FoF, can have various causes, including adverse effects of medicines and subcortical or cortical vascular diseases [14]. Studies also show that FoF can be a marker of cognitive decline [15–17]. It has been shown that FoF can appear before cognitive impairment [15], but also that people with mild cognitive impairment have increased risk to develop FoF [16]. Understanding this bidirectional relationship can promote holistic interventions that address both cognitive and physical health to prevent FoF and support the well-being of older adults.

Despite a link between fall risk and cognition, little attention has been paid to the relationship between FoF and executive functions (EFs). EFs are a set of cognitive functions associated with the frontal lobes of the brain [18], particularly the prefrontal region, with its connections to the thalamus and basal ganglia [19]. EFs include higher-order abilities, such as planning, reasoning, and problem-solving, that enable goal achievement, adaptation to everyday situations, and management of social interactions. Signs of executive dysfunction are poor attention, impulsivity, difficulties with planning and organization, balance problems, and falls [18, 20, 21]. Cognition and mobility are interrelated because of shared neural correlates. EFs are highly involved in safe and effective gait that requires planning, attention, monitoring movements and flexibility to adjust to changes in the environment. Therefore, cognitive decline, especially in EFs, can make individuals more prone to falls [22]. A study involving older adults during dual-task walking, which requires attention and EF, showed that participants with FoF had higher and less efficient prefrontal cortex activation [21], suggesting that FoF might be linked to neural disorganization in this brain area.

In another study, FoF was associated with executive dysfunction in adults over age 50 years [23], but the authors cited limitations, including the need for longer follow-up and for inclusion of more participants over age of 75 years. To our knowledge, no studies of FoF and its connection to EF among people over 80 years are available. To address this gap, we assessed associations of EFs with FoF among people aged 80 years or older, hypothesizing that higher EF would be associated with less FoF. Understanding this possible link might open the way to possible treatment and prevention measures.

## Methods

### Participants and data collection

The Silver-MONICA study is an extension of the Northern Sweden MONICA (MONItoring of trends and determinants in CArdiovascular disease) study [24], initiated by the World Health Organization in the 1980 s. Altogether, eight cross-sectional surveys have been conducted from 1987 to 2022 to track the prevalence of cardiovascular disease and related risk factors. Participants aged 25 to 75 years were randomly selected from the population in two of the northernmost counties in Sweden (Norrbotten and Västerbotten) with a target population of 510,000 people. The Silver-MONICA extension included participants in the 1999 survey who would reach age 80 years or older by 2016 and beyond (*n* = 1595), and surviving respondents in this group were invited to participate in the extension during 2016–2019. Two teams (one in each county) with trained personnel (physiotherapists, nurses, and physicians) visited consenting participants in their homes for data collection. The methodology in Silver-MONICA included elements from the baseline MONICA survey in 1999 [25], combined with geriatric research instruments and scales used in the Umeå85 +/GERDA study (GERontological database) [26]. Individuals who were unable to collaborate because of cognitive impairment were excluded at this stage. Data collection included an interview survey with a predetermined number of questions and assessment scales. In this cross-sectional study, we investigated the association between EFs and FoF among individuals aged 80 years and older, using data collected once for each participant between 2016–2019.

### Measuring EF

The Frontal Assessment Battery (FAB) is a cognitive screening test designed to assess EF and takes approximately 10 min to administer [27]. It has been validated among older adults in several countries, including in Sweden [28–32], and is a reliable screening tool. The FAB consists of six tasks scored 0–3, with higher scores indicating better performance. The maximum possible FAB score is 18, and 12 is the suggested cut-off for dysfunction [30].

The first task (*Similarities*) explores conceptualization and abstract reasoning. In this task, the person is asked to identify the superordinate concept linking two or more objects by indicating in what way they are alike (e.g., two different fruits). Only one category response is considered correct. The second task (*Lexical fluency*) assesses mental flexibility, strategy, and self-organization by asking the person to name as many words as possible with the initial letter “S” in 60 s. The third task (*Motor series*) explores motor programming and planning. The person is asked to copy the test leader in performing the series “fist, edge, palm” with their hand three times and then repeating the series six times on their own. The next task (*Conflicting instructions*) explores sensitivity to interference and self-regulation ability by asking the person to execute one action in response to a different action (knock on the table once when the administrator knocks twice, and knock twice when the administrator knocks once, in a predefined set of knocks). The fifth task (*Go/no-go*) assesses inhibitory control and impulsivity by instructing the participant that when the instructor knocks once, they should also knock once, whereas when the instructor knocks twice, they should do nothing. The last task (*Prehension behavior*) explores the tendency to activate patterns of behavior that are involuntarily triggered by sensory stimuli in the environment, such as grasping objects close at hand. The task involves inhibiting the impulse to grasp the test leader’s hands.

### Measuring FoF

The translated Falls Self-Efficacy Scale–International (FES-I) [33] questionnaire has good psychometric properties in an older population [34]. It contains 16 items scored on a four-point scale based on the level of concern about falling when carrying out various activities (1 = not at all concerned; 4 = very concerned). The concern scores are usually defined as follows: low concern, 16–19 points; moderate concern, 20–27 points; and high concern, 28–64 points [35]. If a participant did not typically perform the activity in question, they were asked to consider how concerned they would be about falling in that hypothetical situation. According to scoring instructions [36], new values were computed for individuals who missed fewer than four items (*n* = 19) (total score divided by the number of items answered multiplied by 16). The majority of these (80%) had only one item missing.

### Characteristics and covariates

Basic participant characteristics are presented for the whole sample and separately for men and women (Table 1). Participant characteristics, e.g. gait speed and depressive symptoms known to be associated with FoF [13] were included in the regression analyses (Table 2). Additionally, analyses were adjusted for variables that have been shown in previous literature to be factors associated with FoF such as a history of stroke [37], pain [38], impaired vision [39], impaired hearing [40], and loneliness [41]. Considered sociodemographic factors were age, sex, and years of education.

**Table 1:** Participant characteristics

Variable	All participants (*N* = 434)	Men (*n*=201)	Women (*n*=233)
*Sociodemographic*			
Sex, n (%)		201 (46.3)	233 (53.7)
Age in years, mean ± SD (range)	84.4 ± 3.4 (79.4–96.3)	84.1 ± 3.5 (79.4–96.3)	84.7 ± 3.34 (79.6–90.5)
Education years, mean ± SD (*n*)	9.5 ± 3.6 (433)	9.57 ± 4.0 (200)	9.36 ± 3.19 (233)
*Physical performance and disability measures*			
Gait speed m/s, mean ± SD (*n*) (range 0.14-1.45)	0.7 ± 0.2 (429)	0.73 ± 0.21 (199)	0.67 ± 0.21 (230)
Mobility aid, *n* (%)	217 (50)	76 (37.8)	141 (60.5)
*Health factors*			
History of stroke, *n* (%)	64 (14.7)	35 (17.4)	29 (12.4)
Diabetes, *n* (%)	81 (18.7)	46 (22.9)	35 (15.0)
Heart failure, *n* (%)	56 (12.9)	25 (12.4)	31 (13.3)
Pain in the last week, *n* (%)	256 (59)	104 (51.7)	152 (65.2)
Impaired vision, *n* (%)	9 (2.1)	3 (1.5)	6 (2.6)
Impaired hearing, *n* (%)	46 (10.6)	28 (13.9)	18 (7.7)
Falls in the past 12 months, *n* (%)	194 (44.7)	93 (46.3)	101 (43.3)
Hip fracture, last 5 years and/or before 5 years ago, *n* (%)	31 (7)	13 (6.4)	18 (7.7)
*Psychological and cognitive*			
FAB, mean ± SD (range 0–18)	12.9 ± 3.2	12.75 ± 2.94	13.06 ± 3.35
FES-I, mean ± SD (range 16–61)	20.65 ± 6.02	19.11 ± 4.53	22.11 ± 6.90
PGCMS, mean ± SD (psychological well-being)	12.8 ± 2.8	13.39 ± 2.52	12.31 ± 2.97
GDS-15, mean ± SD (symptoms of depression)	2.2 ± 1.9	2.14 ± 1.78	2.31 ± 2.04
MMSE, mean ± SD (cognition)	25.6 ± 3.9	25.7 ± 3.40	25.53 ± 4.31
Dementia diagnosis, *n* (%)	112 (25.8)	54 (26.9)	58 (24.9)
Depression diagnosis, *n*/*N* (%)	103/428 (24.1)	36/199 (17.9)	67/229 (28.8)
*Social/environmental*			
Live alone, *n* (%)	216 (49.8)	52 (25.9)	164 (70.4)
Loneliness, *n*/*N* (%)	145/432 (33.6)	42/200 (20.9)	103/232 (44.2)

*Abbreviations*: *FAB* Frontal Assessment Battery, *FES-I* Falls Self-Efficacy Scale International, *GDS-15* Geriatric Depression Scale-15, *MMSE* Mini Mental State Examination, *PGCMS* Philadelphia Geriatric Scale of Morale (0–17), *SD* standard deviation

**Table 2:** Univariable and multivariable linear regression for variables showing an association with FES-I

	**Univariable regression analysis**		**Multivariable regression analysis**	
**Variable**	***β *(95% CI)**	***P* value**	***β *(95% CI)**	***P* value**
FAB	−0.42 (−0.59 to −0.24)	**< 0.001**	**−0.23** (−0.42 to −0.03)	**0.021**
*Sociodemographic*				
Sex (male)	−2.94 (−4.06 to −1.81)	**< 0.001**	**−1.88** (−2.89 to −0.88)	**< 0.001**
Age, years	0.43 (0.27 to 0.60)	** < 0.001**	0.08 (−0.07 to 0.23)	0.295
Education years	−0.08 (−0.24 to 0.08)	0.316	**0.16** (0.02 to 0.30)	**0.026**
*Physical performance and disability measures*				
Gait speed, m/s	−10.22 (−12.54 to −7.91)	**< 0.001**	**−4.73** (−7.48 to −1.98)	**< 0.001**
Mobility aid	4.71 (3.64 to 5.78)	**< 0.001**	**1.30** (0.12 to 2.48)	**0.031**
*Health factors*				
History of stroke	−0.19 (−1.85 to 1.48)	0.825		
Pain in the last week	2.56 (1.41 to 3.71)	**< 0.001**	0.97 (−0.02 to 1.93)	0.050
Impaired vision	0.43 (−3.57 to 4.42)	0.833		
Impaired hearing	1.95 (0.08 to 3.82)	**0.041**	0.34 (−1.26 to 1.94)	0.675
Falls in the past 12 months	2.05 (0.89 to 3.21)	**< 0.001**	0.62 (−0.34 to 1.59)	0.205
*Psychological and cognitive*				
GDS-15, mean (SD) (depressive symptoms)	1.41 (1.14 to 1.68)	**< 0.001**	**0.88** (0.60 to 1.16)	**< 0.001**
MMSE, mean (SD) (cognition)	−0.16 (−0.31 to −0.02)	**0.029**	**0.16** (0.08 to 0.31)	**0.039**
*Social/environmental*				
Loneliness	2.72 (1.54 to 3.90)	**< 0.001**	0.25 (−0.80 to 1.31)	0.637

In the multivariable regression analysis, adjusted R^2^ = 0.302

Variables in univariable regression with *p* <0.15  were added to the multivariable regression analysis. Age, sex, and education years were added regardless of their *p*-value

*Abbreviations: CI* confidence interval, *FAB* Frontal Assessment Battery, *FES-I* Falls Self-Efficacy Scale–International, *GDS-15* Geriatric Depression Scale-15, *MMSE* Mini Mental State Examination

Physical performance included gait speed (m/s) and the use of any mobility aid. Gait speed was captured in terms of preferred gait speed, measured in a 2.4-m walk and taken as the means of two attempts [42]. Use of a walking aid was allowed.

The health factors included self-reported history of stroke, pain in the last week, and falls in the past 12 months. Hearing was assessed and considered impaired when a participant could not hear a conversation in the usual speaking volume from a 1-m distance, with or without hearing aid. Vision was tested and considered impaired when a participant could not read a sentence printed in 5-mm capital letters at a 30-cm distance, with or without glasses. History of falls was assessed by a question concerning the number of falls in the last 12 months and was dichotomized as 0 for no falls and 1 for one or more falls.

Cognitive function was assessed with the Mini-Mental State Examination (MMSE) [43] and symptoms of depression with the Geriatric Depression Scale [44]. The “loneliness” variable was dichotomized from the survey question: Do you feel lonely? Two responses – 1) “often” and 2) “sometimes” – were considered as a “yes,” and two responses – 3) “seldom” and 4) “never” – were considered as a “no.”

### Statistical analyses

Proportions (%) and means for participant characteristics were calculated and categorized as sociodemographic factors, physical performance, and disability measures, health factors, psychological and cognitive factors, and social and environmental variables.

To rule out possible non-linear relationships, we tested spline functions using RStudio (Rstudio Team, 2020). We found that the relationship between FES-I and FAB was linear, so we used SPSS Statistics version 29.0 (IBM SPSS I Armonk, NY: IBM Corp) for all statistical analyses.

In the univariable analyses, linear regression (general linear model) was used to examine the association of FoF (FES-I) and EF (FAB) with other study variables. Multicollinearity was tested with a correlation analysis (Pearson), and no r-values exceeded 0.7, which was considered the limit indicating that an item should be removed. Multicollinearity was further tested with the variance inflation factor, which ranged from 1 upwards. No variable reached more than 1.8, so all variables were considered for the regression model. Variables with a *p*-value < 0.15 in the univariable analysis were included in the linear multivariable regression analysis [45], whereas sex, age, and years of education were included regardless of the *p*-value.

Finally, we examined the correlation (Pearson) between FES-I and items on the FAB.

In all analyses, a *p*-value < 0.05 was considered statistically significant.

## Results

Of 806 eligible participants, 541 individuals aged 80 years or older agreed to participate, and of this group, 483 agreed to a home visit. Participants and non-participants did not differ regarding sex (*p* = 0.34), but non-participants were older (*p* = 0.04). Forty-nine participants could not complete the FAB and/or the FES-I and were excluded. The reasons for inability to complete the scales were poor physical or cognitive ability, blindness, deafness, or lack of motivation.

Finally, 434 participants for whom data on FoF status and EF were available were included (53.7% women, 46.3% men). Their mean age ± standard deviation was 84.4 ± 3.4 years (range 79.4–96.3 years), and 25.8% had a diagnosis of dementia. Baseline characteristics regarding sociodemographic factors, health factors, psychological and cognitive factors, and social and environmental factors are detailed in Table 1.

**Fig. 1:**
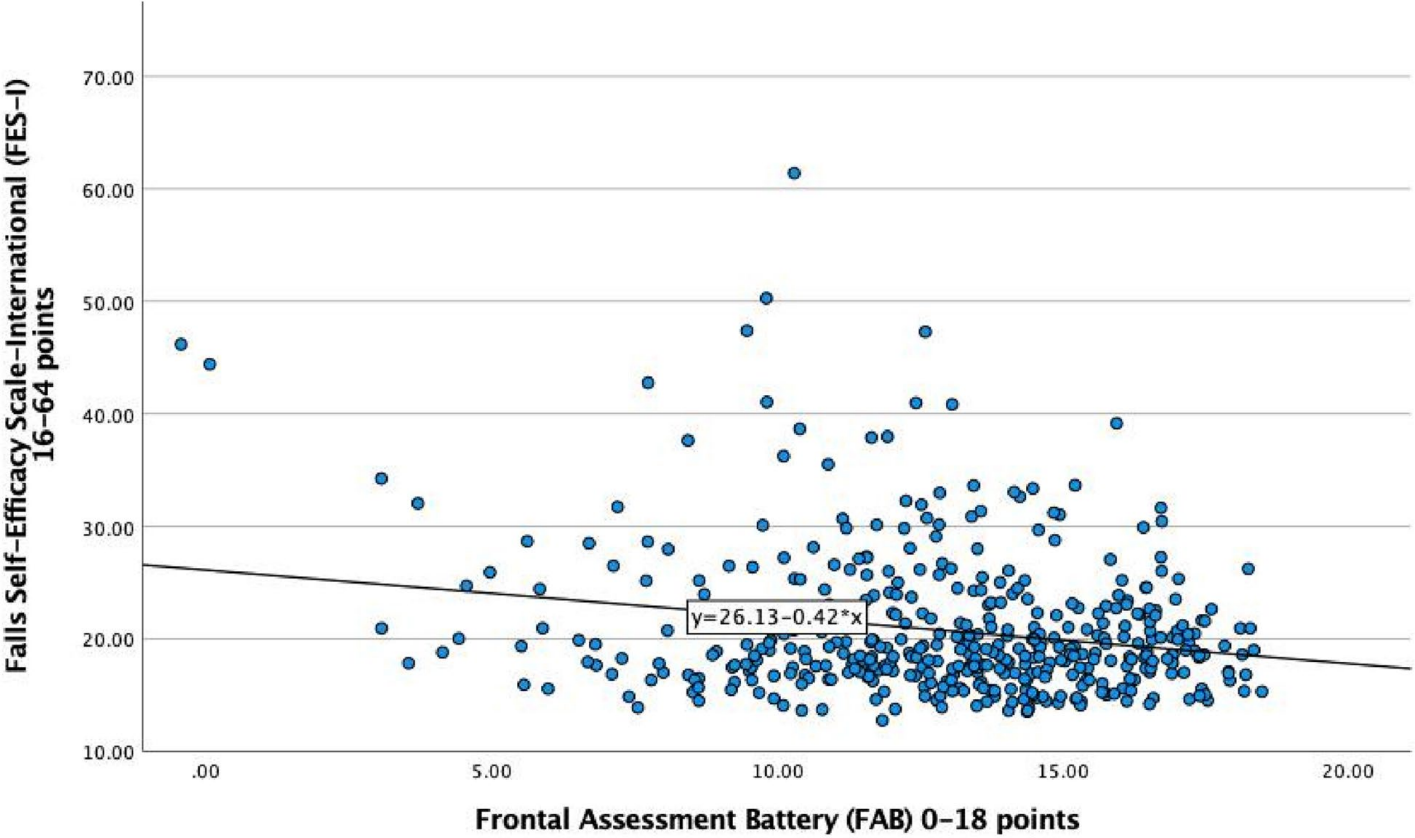
The association between FES-I and the FAB score in 434 participants in the Silver-MONICA study. LEGEND: FAB = Frontal Assessment Battery, FES-I = Falls Self-Efficacy Scale-International. Pearson correlation: R = −0.22, *p* < 0.001

The mean FAB score was 12.9 ± 3.2, and the mean FES-I score was 20.7 ± 6.0 points. In this cohort, 12.5% had high FoF, 29.5% moderate FoF, and 58.0% low FoF. Of those with low FoF there were 107 who reported no FoF. Figure 1 illustrates the association between FES-I (points) and the total FAB score for all participants.

The results from the univariable and multivariable regression analyses for the associations between FES-I and other study variables are shown in Table 2. In the multivariable analysis, a high FAB score was inversely associated with FoF (*β* = −0.23; 95% confidence interval, −0.42 to −0.03; *p* = 0.021), even after adjustment. Other variables associated with higher levels of FoF were female sex, more years of education, slower gait speed, the use of mobility aids, higher MMSE score, and depressive symptoms. This model explained 30.2% of the variance (adjusted R^2^ = 0.302).

The correlation coefficients between the FES-I score and the total FAB score and its subitems are shown in Table 3. The total FAB score was inversely associated (*r* = −0.22, *p* = < 0.001) with FES-I, as well as with the subitems measuring lexical fluency, inhibitory control, sustained attention, self-organization, motor programming, and planning. However, the first subitem, “similarities,” was not associated with FES-1.

**Table 3:** Correlation (Pearson) between fear of falling and executive function

Variable	FES-I total	
	*N* = 434	
	Correlation coefficient	*P*-value
FAB total	−0.22	**< 0.001**
FAB item 1: Similarities	−0.04	0.360
FAB item 2: Lexical fluency	−0.15	**0.002**
FAB item 3: Motor series	−0.13	**0.005**
FAB item 4: Conflicting instructions	−0.13	**0.007**
FAB item 5: Go/no-go	−0.18	**< 0.001**
FAB item 6: Prehension behavior	−0.17	**< 0.001**

*FAB* Frontal Assessment Battery, *FES-I* Falls Self-Efficacy Scale–International

## Discussion

Among people aged 80 years or older, we found an inverse association between EFs measured with the FAB and FoF measured with FES-I. Our results suggest that lower levels of EFs are associated with greater FoF, and higher levels of EFs are associated with lower FoF, even with adjustment for other relevant factors. The findings support our hypothesis that higher EF is associated with less FoF, and are consistent with previous results in a younger, healthier group of participants [23]. To our knowledge, these findings are the first indicator that an inverse association between EFs and FoF also exists among people aged 80 years or older.

To deepen understanding of this association, we analyzed FAB subitems and found weak but significant correlations between FES-I and five subitems. The specific EFs that seemed to be important were inhibitory control, lexical fluency (sustained attention), self-organization, motor programming, and planning. Unfortunately, there is a lack of studies exploring the association between FoF and these specific functions among people aged 80 and over. Theoretically, impairment might affect FoF in various ways. Limited inhibitory control can make it difficult to stop unsafe movements which increases fall risk [46], and thereby possibly also FoF. The ability to stop automatic behaviors in daily life is critical, for instance preventing a step from landing on a slippery surface.

Interestingly, the verbal task “Lexical fluency” correlated with FoF, which was unexpected for a verbal task but aligns with findings from the study mentioned above that involved younger participants [23]. However, the other verbal subtask, “Similarities,” did not show a correlation with FAB. We interpret this difference as indicating that, unlike “Similarities”, the “Lexical fluency” task requires sustained attention, which can be important in FoF and fall risk. Higher sustained attention could also indicate better physical performance, potentially reducing FoF.

It can be argued that concerns about falling are reasonable for people over 80, as their physical abilities can be compromised and the consequences more severe compared with falls in younger people. Concerns about falling may indeed reflect adequate consequence thinking. In older age, however, physical exercise becomes increasingly important to maintain muscle strength and balance to prevent falls, and for this reason alone, FoF should be prevented and addressed as much as possible.

To evaluate whether FoF is adaptive or maladaptive may require consideration of an individual’s physiological fall risk. Delbaere et al. [35] investigated adults aged 70–90 years (*n* = 500) and found that, among those with a low physiological fall risk, a high perceived fall risk was associated with lower EF and an increased probability of falling. This pattern implies that the actual fall risk must be considered in assessing whether FoF is adaptive or maladaptive and that the association between FoF and EF may be stronger among those with maladaptive FoF.

In a recent review, Ellmers et al. [12] presented a new conceptual framework (the Perceived Control Model of Falling) and a tool (the Updated Perceived Control over Falling Scale) intended to help clinicians distinguish between maladaptive FoF that is likely to increase fall risk and when FoF might be protective. The concept of “perceived control,” which reflects a person’s perceived ability to avoid a fall in a certain situation, was identified as a key factor in whether FoF is protective or maladaptive. Interestingly, in this study, we observed that 107 individuals reported no FoF (not even when walking on slippery surfaces), which is unexpected for individuals aged 80 and over. Sometimes both FoF or the absence of FoF can be maladaptive. For instance, individuals may experience too much FoF when the risk of falling is low or too little FoF when the risk of falling is high. This would require more in-depth investigation that our study was not designed to explore. Nevertheless, we acknowledge the importance of this distinction for future research in understanding the complex association between FoF and its consequences. We argue that EFs are relevant to this ability to adapt and manage challenging situations in everyday life and at the same time prevent FoF from becoming overwhelming.

Other factors associated with FoF were female sex, more education years, slower gait speed, the use of mobility aids, higher MMSE score, and symptoms of depression. The link between FoF and symptoms of depression was not surprising given the frequently identified association of the two among older adults [13]. Depression was not the focus of this study, so we did not attempt further analyses here.

Notably, while our primary focus was on FAB, we noticed that increased years of education and higher MMSE significantly increased FoF in the multivariable regression but was inversely associated in the univariable regression. An interpretation can be that higher education or better MMSE might be associated with a greater awareness of fall risks, leading to increased fear in this vulnerable age group. Since this was not the case for FAB, we interpreted that the decline in EFs is of particular importance to FoF. This aligns with a recent study [47] which showed that reduction in visuospatial and frontal or executive functions may be more strongly linked to FoF than other cognitive domains.

Among our participants, FoF prevalence was moderate to high at 42%, indicating that it is a common problem among the oldest people. Our results suggest that evaluation and treatment of FoF among people aged 80 years or older should target EFs as well as physical performance and symptoms of depression.

### Future studies

When evaluating the association between EF and FoF among people aged 80 years or older, future studies should include measures of physiological fall risk and perceived control to understand whether FoF is protective or maladaptive. Also, of interest is assessing other aspects of EF, such as judgment and self-awareness, that the FAB does not. Furthermore, studies should assess whether EF training could be valuable in treating and preventing FoF among people at the most advanced ages. According to Barban et al. [48], intervention programs combining balance and strength training with cognitive training of EF might be helpful. Our results suggest that specific training in inhibitory control, sustained attention, self-organization, motor programming, and planning should be further investigated.

### Strengths and limitations

This study is the first to assess EF and FoF among people aged 80 or older. A strength of the study is the participant selection, with the inclusion of surviving participants from the population-based, randomized MONICA study. However, because not all invited participants accepted the invitation and some could not complete all the questions and tasks, selection bias towards participants with better EF is likely. Indeed, dropout analysis showed that non-participants were older than participants, suggesting that the negative association of cognitive function and EF with FoF may be even greater than reported here. We nevertheless cannot exclude residual confounding from unmeasured variables, for instance psychotropic medications that were not included in our current data set.

Another strength is the design of the study, with home visits, actual interaction with participants, and the opportunity for a more holistic assessment with clinical observations than registry data allow. The arrangement with home visits, including those living in group care, was crucial for including as many participants as possible regardless of individual health and functional status. In this study, 25.8% of participants had a dementia diagnosis. Including participants with low functional status and dementia increased the representativeness of our cohort.

However, cognitive dysfunction could have affected the validity of self-assessment forms such as FES-I. For instance, participants who did not engage in the various activities covered by the FES-I were asked how concerned they would be if they performed the activity. Such questions require abstract thinking, which can be difficult for those with affected cognitive abilities. Therefore, the broad inclusion criteria and heterogeneity of our study participants could be a methodological challenge in evaluations of FoF among those in the most advanced age group. Finally, this study is cross-sectional, which limits our ability to draw conclusions about causality. As data were collected at a single point in time, we cannot determine whether lower levels of EFs lead to increased FoF, or vice versa.

## Conclusions

Our findings add to evidence of an inverse association between EFs and FoF, with lower EF linked to higher FoF among people aged 80 years or older. The specific EFs that seemed to be important were inhibitory control, sustained attention, self-organization, motor programming, and planning. This information is important for treating and preventing FoF in this population.

## Data Availability

The data that support the findings of this study are not openly available due to reasons of sensitivity and are available from the Section of Biobank and Registry Support at Umeå University (info.brs@umu.se) upon reasonable request. Data are located in controlled access data storage at Umeå University.

## Abbreviations

EF: Executive function
EFs: Executive functions
FoF: Fear of falling
FAB: Frontal Assessment Battery
FES-I: Falls Self Efficacy Scale - International
MMSE: Mini Mental State Examination
GDS-15: Geriatric Depression Scale-15
PGCMS: Philadelphia Geriatric Center Morale Scale
